# Endoscopic Ultrasound-Guided Biliary Drainage Using a Fully Covered Metallic Stent after Failed Endoscopic Retrograde Cholangiopancreatography

**DOI:** 10.1155/2016/9469472

**Published:** 2016-07-31

**Authors:** Jintao Guo, Siyu Sun, Xiang Liu, Sheng Wang, Nan Ge, Guoxin Wang

**Affiliations:** Shengjing Hospital of China Medical University, Shenyang 110004, China

## Abstract

*Background and Study Aims.* Endoscopic ultrasound- (EUS-) guided biliary drainage (EUS-BD) is an alternative treatment for biliary obstruction after failed endoscopic retrograde cholangiopancreatography (ERCP). In this study, we present the outcomes of inpatients with obstructive jaundice treated with EUS-BD using a fully covered metallic stent after failed ERCP.* Patients and Methods.* A total of 21 patients with biliary obstruction due to malignant tumors and prior unsuccessful ERCP underwent EUS via an intra- or extrahepatic approach with fully covered metallic stent between March 2014 and October 2015. A single endoscopist performed all procedures.* Results.* Seven patients underwent hepatogastrostomy (HGS) and 14 underwent choledochoduodenostomy (CDS). The technical and clinical success rates were both 100%. There was no difference in efficacy between HGS and CDS. Adverse events occurred in three patients, including two in the HGS group (1 bile leakage and 1 sepsis) and one in the CDS group (sepsis). Four patients died as a result of their primary tumors during a median follow-up period of 13 months (range: 3–21 months). No patient presented with stent migration.* Conclusion.* EUS-BD using a fully covered metallic stent appears to be a safe and effective method for the treatment of obstructive jaundice.

## 1. Introduction

Endoscopic retrograde cholangiopancreatography (ERCP) has become the first-line therapy for bile duct drainage [1, 2]. In the hands of experienced endoscopists, conventional ERCP results in a failed-cannulation rate of only 3%–5%. Most failures are associated with altered anatomy (e.g., because of previous surgery such as surgical bypass, gastrectomy or Whipple resection) or technical difficulties related to duodenal or biliary obstruction [3]. Percutaneous or surgical interventions are mandatory in patients with failed ERCP but are associated with considerable morbidity and mortality [4, 5]. Interventional EUS is a minimally invasive procedure, and EUS-BD has recently been developed as a salvage therapy for transpapillary treatment [6–9]. The first case reports of EUS-guided transgastric (hepatogastrostomy, HGS) and transduodenal (choledochoduodenostomy, CDS) biliary drainage using plastic or metallic stents were published in the early 2000s [10, 11], followed by subsequent case series [12–16].

The current study aimed to report the outcomes of EUS-BD using a fully covered metallic stent for the treatment of 21 patients with obstructive jaundice and failed ERCP.

## 2. Patients and Methods

### 2.1. Patients

All patients who presented with obstructive jaundice and underwent EUS-BD with placement of a fully covered metallic stent after failed ERCP were entered into the study. A total of 45 patients suffered from obstructive jaundice and underwent failed ERCP in our endoscopy center from March 2014 to October 2015. Sixteen of these patients underwent percutaneous transhepatic biliary drainage (PTBD) and eight underwent surgery. The remaining 21 patients underwent EUS-BD and were included in the current study.

This study was approved by the Institutional Review Board and Ethics Committee of China Medical University. All patients chose their therapeutic course voluntarily and provided written informed consent for their participation in this study. All drainage procedures were performed by the same endoscopist who was familiar with interventional EUS techniques.

### 2.2. Procedures

The equipment used included a linear array echoendoscope (EG3830UT; Pentax, Tokyo, Japan) with an adjustable ultrasonic frequency of 5, 7.5, or 10 MHz, in combination with an ultrasound scanner (EUB 6500; Hitachi, Tokyo, Japan). 19-gauge needle (EUS N-19-T; Wilson-Cook Medical, Winston-Salem, NC, USA) was used for puncture. A 0.035-inch guidewire (Jagwire; Boston-Scientific, Natick, MA, USA) was used for guidance. A cystotome (10 Fr; Wilson-Cook Medical) was used to dilate the tract and create a large fistula. A fully covered metallic stent (Wilson-Cook Medical, Winston-Salem, NC, USA) was used for biliary drainage. Prophylactic intravenous antibiotics (ceftriaxone, 1 g) were administered routinely twice daily for at least 2 days after the procedure.

### 2.3. EUS-Guided HGS (EUS-HGS) (Figure 1)

EUS-HGS was usually performed in patients who suffered from proximal bile duct obstruction, surgically altered anatomy, or duodenal-bulb invasion.

The intrahepatic approach was performed via the neighboring gastrointestinal tract to allow visualization of the left intrahepatic bile ducts. The usual puncture point was in the cardia or the lesser curvature of the stomach. The echoendoscope was advanced into the stomach. After checking the local vasculature by color Doppler, the 19 G EUS puncture needle was then advanced into the intrahepatic duct and cholangiography was performed, which usually delineated the dilated biliary tree down to the point of obstruction. A guidewire was then inserted through the needle and a cystotome was used to create a fistula between the stomach (or jejunum in patients with total gastrectomy) and the left hepatic duct. Once the fistula had been dilated, a fully covered self-expandable metal stent (SEMS) (8–10 diameter × 4–10 cm long, fully covered with a silicon membrane) was inserted and deployed transmurally. To avoid bile leakage into the peritoneum, a 7 Fr nasobiliary catheter was sometimes placed through the metallic stent for 48 h. Sometimes an uncovered SEMS was placed through the covered stent to avoid stent migration.

### 2.4. EUS-Guided CDS (EUS-CDS) (Figure 2)

EUS-CDS was usually performed in patients who suffered from mid or distal bile duct obstruction or insufficient intrahepatic bile duct dilatation.

For CDS, the needle was directed towards the hilar (proximal) bile duct by maintaining a long scope position, usually from the duodenal bulb. This was important because an upward needle orientation facilitated the procedure by decreasing the angle for transmural stent advancement into the bile duct. After placement of a guidewire, a fistula was created using a cystotome, to pass the stent into the bile duct. Once the fistula was dilated, an SEMS (fully covered) was inserted and deployed transmurally.

## 3. Results

A total of 21 patients (15 male, 6 female; mean age 67 years, range 41–79 years) were included in the study. The biliary obstruction was malignant in all cases. The reasons for failed ERCP were duodenum stenosis (*n* = 9), surgical bypass (*n* = 1), biliary cannulation failure associated with periampullary tumor infiltration (*n* = 10), and altered position of the ampulla (*n* = 1). The causes of duodenal stenosis were pancreatic carcinoma (*n* = 4), ampullary carcinoma (*n* = 3), and duodenal carcinoma (*n* = 2). Surgical bypass was associated with a previous gastrectomy.

Seven patients underwent HGS and 14 underwent CDS. Both the technical and the clinical success rates were 100%. Bilirubin levels fell in all patients after the procedure. There was no difference in efficacy between HGS and CDS. Adverse events occurred in three patients, including two in the HGS group (1 bile leakage and 1 sepsis) and one in the CDS group (sepsis). A 7 Fr nasobiliary catheter was placed through the metallic stent in the two patients who suffered sepsis, after which the body temperature of both patients returned to normal within 48 h. An uncovered SEMS was placed through the covered stent in one case to avoid stent migration.

Patients were followed up for a mean of 13 months (range: 3–21 months). During this period, four patients died as a result of their primary tumors, two patients presented with stent occlusion, and successful recanalization was achieved in both patients. No patient presented with stent migration.

## 4. Discussion

Technologic advances in echoendoscopes, processors, and accessories have allowed EUS to progress from a largely diagnostic to a therapeutic modality [17]. The widespread adoption of minimally invasive surgery and radiologic procedures has led to an increase in the use of therapeutic EUS for the curative and/or palliative treatment of gastrointestinal and pancreaticobiliary diseases [17].

Endoscopic retrograde cholangiography with BD remains the most frequent method for palliation of malignant biliary obstruction, with cases of ERCP failure traditionally being referred for either PTBD or surgery. However, both PTBD and surgery have relatively high complication rates, which, together with patient dissatisfaction associated with external drainage, make these options undesirable. Khashab et al. [18] compared the efficacy, safety, and cost of EUS-BD and PTBD in jaundiced patients with distal malignant biliary obstruction after failed ERCP. A total of 73 patients with failed ERCP subsequently underwent either EUS-BD (*n* = 22) or PTBD (*n* = 51), and although the clinical success rates were equivalent (92.2% versus 86.4%, *P* = 0.40), EUS-BD was associated with fewer adverse events (18.2% versus 39.2%) and lower total costs.

EUS-BD has emerged as an effective alternative over the last decade, with significant potential as a minimally invasive and low-risk method of biliary access. Since 2008, numerous studies on EUS-BD have reported high technical and functional success rates and adverse event rates of 3%–23% [6, 7, 9, 19–27].

Complications after EUS-BD include pneumoperitoneum, bile leakage, cholangitis, bleeding, abdominal pain, and stent occlusion. Gupta et al. [28] compared the complication rates of EUS-BD in patients with benign and malignant diseases and found similar complication rates in both groups (26.7% versus 37.1%). They placed stents in 173 patients with malignant etiologies, including 42 (24%) plastic and 131 (76%) metal stents, and found no significant difference in complication rates between the two types of stents but did note a trend towards better outcomes in patients with metal stents (*P* = 0.09).

EUS-BD was initially largely performed using plastic stents, though many experts reported favorable outcomes with SEMS, instead of plastic stents [29–31]. Song et al. [32] performed a study in 15 patients with distal malignant biliary obstruction who were candidates for alternative techniques of biliary decompression following failed ERCP. They achieved a technical success rate following EUS-CDS with a fully covered SEMS of 86.7% (13/15), and a functional success rate of 100% (13/13). Eum et al. [30] studied three consecutive patients who underwent EUS-BD with a fully covered SEMS for biliary decompression and concluded that this technique was able to achieve a large-diameter sustainable fistula. Endoscopic intervention through this fistula thus seems to be feasible and useful for the management of intrabiliary lesions. Fabbri et al. [20] successfully used a new partially covered biliary stent for EUS-assisted cholangiography in patients with malignant biliary obstruction. There were no major complications or procedure-related deaths, and no patients required endoscopic reintervention during the 170-day follow-up period.

We used fully covered SEMS in the current study. These stents may decrease the risk of bile leakage and pneumoperitoneum. Indeed, only one patient suffered from bile leakage (4.8%, 1/21), which occurred in the primary stage of treatment and may have been related to lack of experience of the procedure. The resulting peritonitis was mild and self-limited.

CDS or HGS is used for gastrointestinal luminal access, depending on the desired site. In our study, EUS-HGS was usually performed in patients suffering from proximal bile duct obstruction, surgically altered anatomy, or duodenal-bulb invasion. We found no difference in efficacy between HGS and CDS. Artifon et al. [22] compared the outcomes of EUS-CDS and EUS-HGS in 49 patients with unresectable distal malignant biliary obstruction and failed ERCP. The technical success rates for HGS and CDS were 96% and 91%, and the clinical success rates were 91% and 77%, respectively. The mean procedural times were 47.8 min for HPG and 48.8 min for CDS. The mean quality of life scores were similar during follow-up. They therefore concluded that HGS and CDS were similar in terms of efficacy and safety.

EUS-guided rendezvous is a choice for the patient after failed ERCP with issues of biliary cannulation at the papilla. Compared with direct transluminal techniques, the process of rendezvous is relatively complex and time consuming. In the study of Khashab et al. [6], 35 patients underwent EUS-BD (rendezvous *n* = 13, transluminal *n* = 20). Technical success was achieved in 33 patients (94%), and clinical success was attained in 32 of 33 patients (97.0%). There was no significant difference in adverse event rate between rendezvous and transluminal groups (15.4% versus 10%; *P* = 0.64). In their study, both rendezvous and direct transluminal techniques seem to be equally effective and safe. So, we always choose direct transluminal techniques instead.

In the current study, four of the 21 patients died after about 13 months of follow-up; however, the fully covered SEMS was still functioning after the time span it would be expected to remain in patients with unresectable malignancies.

One disadvantage of fully covered SEMS is their greater cost, compared with plastic stents. However, their long-term patency and significantly lower reintervention rates suggest that metal stents may still represent a cost-effective choice.

In summary, EUS-BD with fully covered SEMS offers great potential as an alternative method of biliary decompression, associated with high success rates, low complication rates, and a lack of fatalities. The present study was limited by its retrospective nature and relatively small sample size. Larger prospective studies are thus needed to confirm these results.

## Figures and Tables

**Figure 1:**
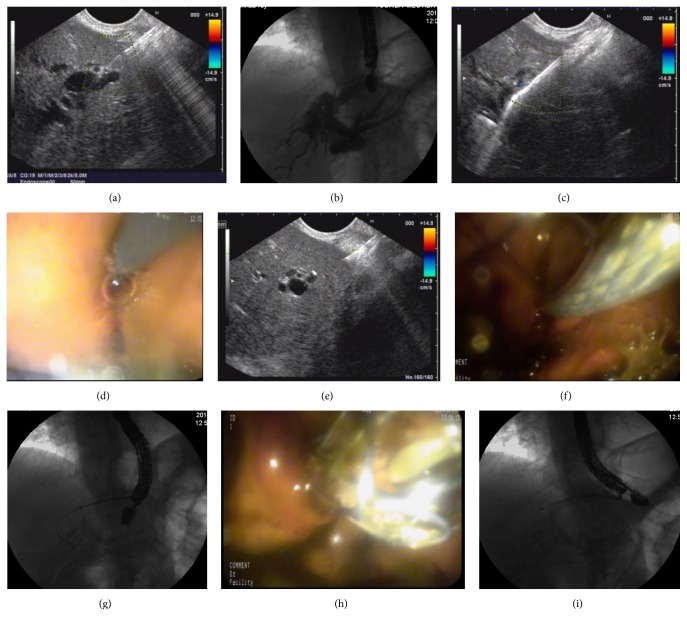
(a) Echoendoscope was advanced into the stomach. After checking local vasculature with color Doppler, the EUS puncture needle was then advanced into the intrahepatic duct. (b) Cholangiography was performed, which usually delineates the dilated biliary tree down to the point of obstruction. (c) A guidewire was then inserted through the needle. (d) The cystotome was used to create a fistula between the stomach and the left hepatic duct. (e) The distance between the stomach and the left hepatic duct was measured. (f)–(i) Once the fistula has been dilated, a fully covered SEMS (10 mm diameter × 8 cm length, fully covered with a silicon membrane) was inserted and deployed transmurally.

**Figure 2:**
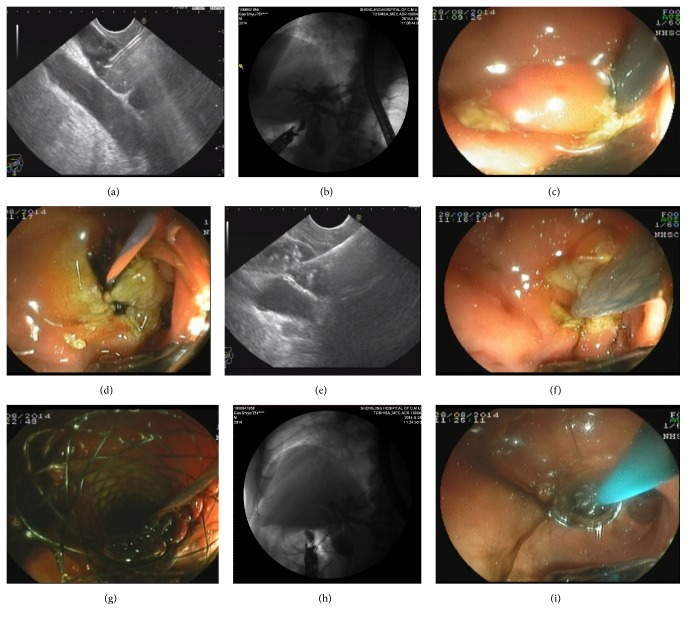
(a) Echoendoscope was advanced into the duodenal bulb. After checking local vasculature with color Doppler, the EUS puncture needle was then advanced into the intrahepatic duct. (b) Cholangiography was performed, which usually delineates the dilated biliary tree down to the point of obstruction. (c) The cystotome was used to create a fistula between the stomach and the left hepatic duct. (d) The puncture site after dilation. (e) The guidewire was observed under the EUS. (f)–(h) The fully covered SEMS was inserted and deployed transmurally. (i) To avoid bile leakage into the peritoneum, a 7 Fr nasobiliary was placed through the metallic stent.
